# Supplementary material for: Yiai Fuzheng decoction inhibits triple-negative breast cancer by remodeling the immune microenvironment
Source: Front Immunol. 2025 Sep 30;16:1615631. doi: 10.3389/fimmu.2025.1615631 (PMC12518410; doi:10.3389/fimmu.2025.1615631)
Supplement: Supplementary file 2 [file DataSheet1.zip › Supplementary File 2/Treatment/C_vs_H/01_Basic_Statistics/Metabolite_Class_Statistics/Class_Pieplot.pdf]

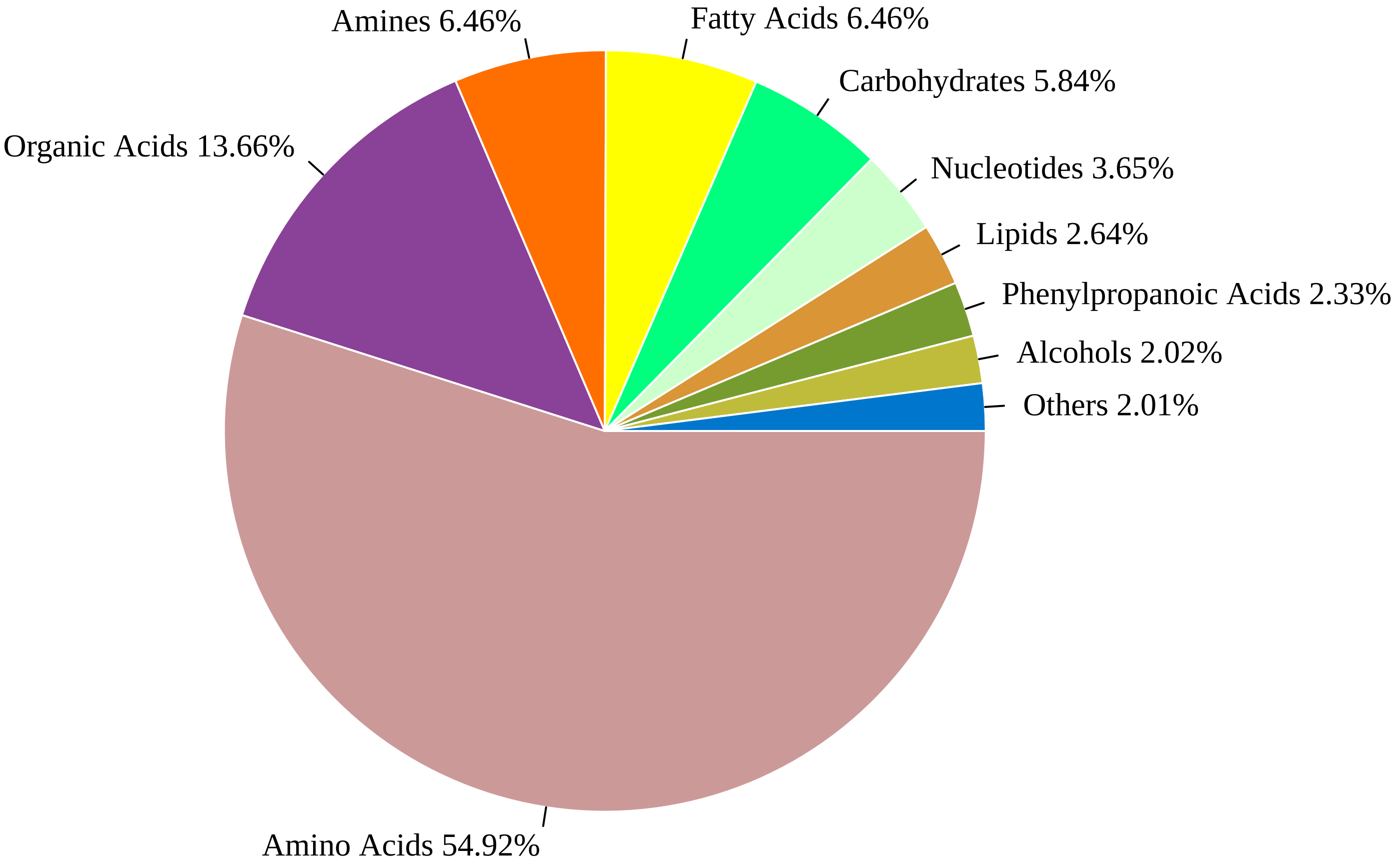
Amines 6.46%
Fatty Acids 6.46%
Carbohydrates 5.84%
Organic Acids 13.66%
Nucleotides 3.65%
Lipids 2.64%
Phenylpropanoic Acids 2.33%
Alcohols 2.02%
Others 2.01%
Amino Acids 54.92%
